# Publication trends and hotspots of drug resistance in colorectal cancer during 2002-2021: A bibliometric and visualized analysis

**DOI:** 10.3389/fonc.2022.947658

**Published:** 2022-08-30

**Authors:** Peng-yue Zhao, Ya-nan Jiao, Zhao-fu Ma, Yang Yan, Yu-xuan Li, Shi-dong Hu, Song-yan Li, Xiao-hui Du

**Affiliations:** Department of General Surgery, First Medical Center of Chinese, People's Liberation Army (PLA) General Hospital, Beijing, China

**Keywords:** drug resistance, colorectal cancer, bibliometric analysis, microRNA, EMT

## Abstract

**Background:**

Chemotherapy, radiotherapy, targeted therapy and immunotherapy have demonstrated expected clinical efficacy, while drug resistance remains the predominant limiting factor to therapeutic failure in patients with colorectal cancer (CRC). Although there have been numerous basic and clinical studies on CRC resistance in recent years, few publications utilized the bibliometric method to evaluate this field. The objective of current study was to provide a comprehensive analysis of the current state and changing trends of drug resistance in CRC over the past 20 years.

**Methods:**

The Web of Science Core Collection (WOSCC) was utilized to extracted all studies regarding drug resistance in CRC during 2002-2021. CiteSpace and online platform of bibliometrics were used to evaluate the contributions of various countries/regions, institutions, authors and journals in this field. Moreover, the recent research hotspots and promising future trends were identified through keywords analysis by CiteSpace and VOSviewer.

**Results:**

1451 related publications from 2002 to 2021 in total were identified and collected. The number of global publications in this field has increased annually. China and the USA occupied the top two places with respect to the number of publications, contributing more than 60% of global publications. Sun Yat-sen University and *Oncotarget* were the institution and journal which published the most papers, respectively. Bardelli A from Italy was the most prolific writer and had the highest H-index. Keywords burst analysis identified that “Growth factor receptor”, “induced apoptosis” and “panitumumab” were the ones with higher burst strength in the early stage of this field. Analysis of keyword emergence time showed that “oxaliplatin resistance”, “MicroRNA” and “epithelial mesenchymal transition (EMT)” were the keywords with later average appearing year (AAY).

**Conclusions:**

The number of publications and research interest on drug resistance in CRC have been increasing annually. The USA and China were the main driver and professor Bardelli A was the most outstanding researcher in this field. Previous studies have mainly concentrated on growth factor receptor and induced apoptosis. Oxaliplatin resistance, microRNA and EMT as recently appeared frontiers of research that should be closely tracked in the future.

## Background

CRC ranks third and second in morbidity and mortality, respectively, and is a heavy burden on global health and medical services (1, 2). The latest global cancer statistics indicated that there were 1.9 million new CRC cases and 935,000 reported deaths every year (1). Noteworthily, the advent of chemotherapy, radiotherapy, targeted therapy and immunotherapy significantly improved the prognosis of CRC patients. However, both intrinsic and acquired mechanisms of drug resistance are commonplace, which remains the principal limiting factor to therapeutic failure in patients with CRC. Similar to the field of infection, intrinsic drug resistance in cancer refers to the innate physiological characteristics of cancer cells that are insensitive to certain anticancer drugs, while acquired drug resistance means that cancer cells exhibited the drug resistance by altering their own genetic materials or attaining exogenous resistance genes during the process of anticancer therapy (3). Therefore, pioneering or revolutionary research to overcome the drug resistance in CRC patients is eagerly desired.

A body of studies have elucidated that the mechanisms by which cancer cells manifest intrinsic and acquired drug resistance are primarily ascribable to the influence of cancer cells on the drug molecule itself or the targets of the drug (4). It is widely recognized that the mechanisms of drug resistance in CRC included increased drug efflux, reduced drug uptake, drug inactivation, target mutation, signaling pathway alterations, apoptosis defects, phenotype switching and so on (4). For instance, the protein ATP-binding cassette (ABC) can act as a transporter to transport multidrug resistance proteins (MRPs), and the latter can act as cell membrane pumps to effectively remove intracellular drugs from cancer cells (5). Previous studies have confirmed that overexpression of these transport proteins is closely associated with poor prognosis in patients with a variety of cancers. Therefore, targeting these transport proteins may be one of the effective approaches to overcome drug resistance in CRC (6). Of note, the coexistence of multiple biological determinants in drug resistance has it a refractory problem in cancer treatment. Fortunately, clinical and scientific researchers have not stopped trying to conquer this dilemma. For instance, early diagnosis and radical removal of cancers, optimization of drug dose, compatibility and time to achieve deeper response, and whole-process monitoring of drug response and timely intervention provide foreseeable hope for the goal of preventing, delaying or even reversing drug resistance in cancer (7).

Although there are numerous studies related to drug resistance in CRC, the diversity and complications of these studies may also bring several issues to relevant researchers. Fortunately, the advent of bibliometrics may provide crucial support for summarizing the research hotspots and predicting the future research direction in a certain field. Bibliometrics is a tool that allows for the qualitative and quantitative analysis of knowledge carriers such as books and literature (8). Bibliometric analysis provides an opportunity to analyze changing trends in a particular field on a global scale and to investigate the contribution of countries/regions, institutions, scholars, etc. (9) What’s more, bibliometric can also analyze the research hotspots in the field and forecast future research directions (10).

To perform a comprehensive analysis of the publications about drug resistance in CRC, the bibliometric method was applied to analyze the overall trends of publications in this field over the past 20 years and to predict possible future research hotspots. We hope that it can serve as a reference for researchers and to provide some clues for future research.

## Methods

### Data sources and search strategies

**Figure 1:**
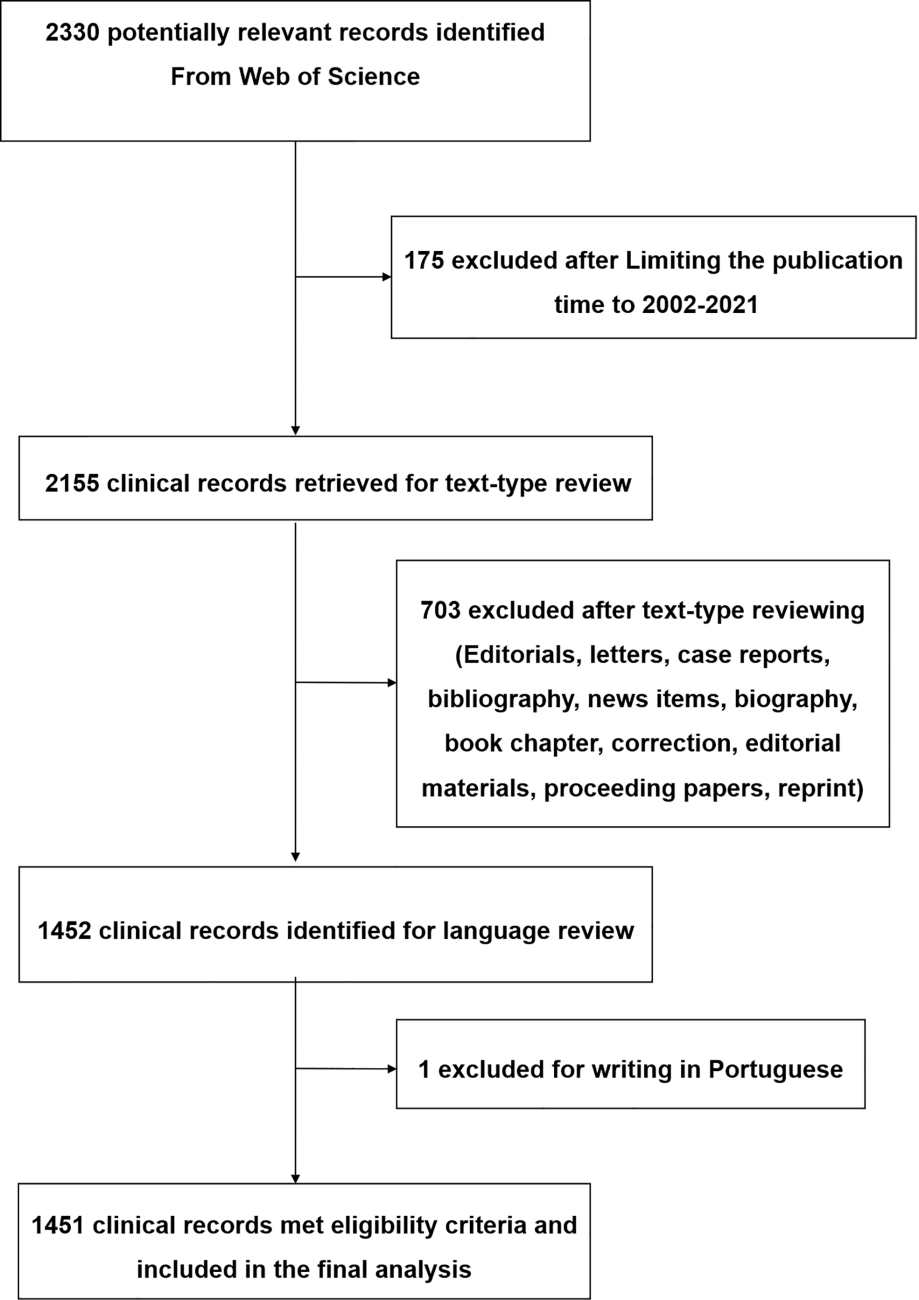
Flow diagram of research selection and screening.

The Science Citation Index-Expanded (SCI-E) is the database that is most commonly used in bibliometric analysis (9, 11). A comprehensive search of the literature was carried out through the WOSCC and the search strategy was as follows: TI = ((resist*) OR (drug resist*) OR (medicine resist*) OR (agent resist*) OR (Antineoplastic Agent resist*) OR (Antineoplastic Drug resist*) OR (Antineoplastic resist*)) AND TI = ((Rectal Neoplasm*) OR (Rectal Tumor*) OR (Rectal Cancer*) OR (Rectum Neoplasm*) OR (Rectum Cancer*) OR (Cancer of the Rectum) OR (Cancer of Rectum) OR (Colorectal Neoplasm*) OR (Colorectal Tumor*) OR (Colorectal Cancer*) OR (Colorectal Carcinoma*) OR (Colonic Neoplasm*) OR (Colon Neoplasm*) OR (Cancer of Colon) OR (Colon Cancer*) OR (Cancer of the Colon) OR (Colonic Cancer*)). The inclusion criteria included: the publication time was from 2002 to 2021; article types were limited to only original articles and reviews; and article language was limited to English. To avoid bias arising from the frequent renewal of the database, all data retrieval and collection were accomplished within one day of 30 April 2022. **Figure 1** illustrates the process of publication enrollment and screening in detail.

### Data collection

Literature screening and data filtering were carried out by two reviewers (P-YZ and Y-NJ) independently. Data including the title, keywords, date of publication, authors, institutions, country or region, journals, total citations and H-index that were obtained from WOSCC. Qualitative and quantitative analyses were conducted by CiteSpace (Drexel University, USA), VOSviewer (Leiden University, Leiden, Netherlands), Microsoft Excel 2016 (Redmond, Washington, USA) and the online platform of bibliometrics (http://bibliometric.com/).

### Bibliometric analysis

All publication characteristics of eligible literature in WOSCC were well documented and described. We gained access to the latest impact factors (IF) of the relevant periodicals by surveying the current edition of JCR (Journal Citation Reports), which is an essential criterion for the evaluation of academic influences (12). The H-index acquired from WOSCC has been widely accepted for evaluating the scientific contribution of a scholar or a country/region. It is defined as H papers that have been published by a scholar or a country/region and each paper has been cited at least H times, including self-citations (13). The number of annual publications and changing trends in different countries/regions were analyzed utilizing the online bibliometrics platform and Microsoft Excel. The relative novelty of keywords was measured by AAY with VOSviewer. AAY refers to the average time calculated by integrating the time of the first occurrence and the last occurrence of a certain keyword, which can intuitively reflect the time of the emergence of the keyword in the early or late and the novelty degree. Co-citation analyses on countries/regions, institutions and journals as well as co-occurrence analyses on keywords were performed by CiteSpace. Additionally, the top strongest citation bursts of institutions, journals and keywords were also derived from this software.

The specific parameter settings of CiteSpace included that time-slicing was from 2002 to 2021 and one year per slice, selection criteria (g-index, g^2^ ≤ k∑_i ≤ g_ c_i_, k ∈ Z^+^, k = 25; Top 50; Top 10%) (11). The specific parameter settings of VOSviewer were illuminated in corresponding figure legends.

## Results

**Figure 2:**
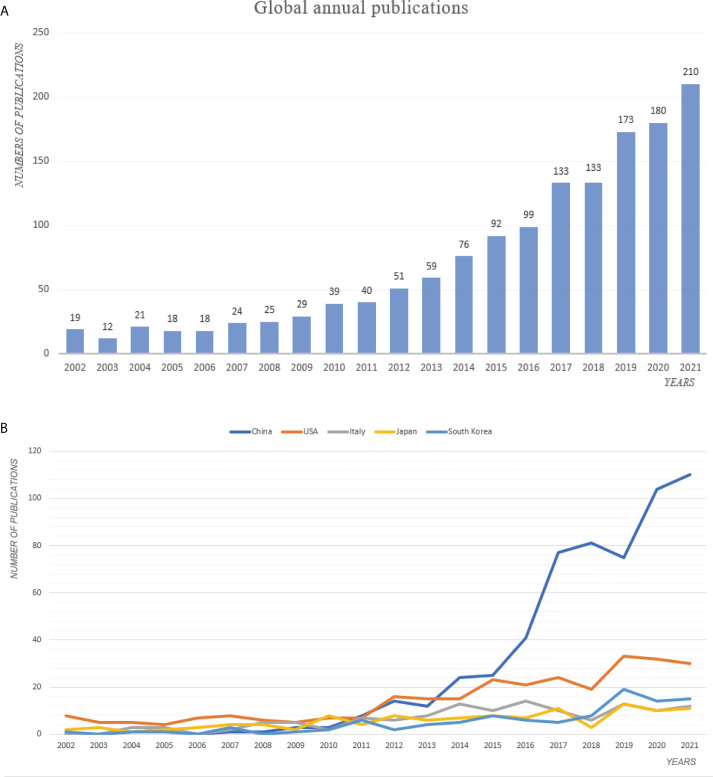
The number of annual publications on drug resistance and colorectal cancer from 2002 to 2021. **(A)** Global; **(B)** Top5 Countries or regions.

A total of 1451 studies published from 2002 to 2021 were enrolled based on the inclusion criteria. The global trend in the number of publications illustrated in **Figure 2A** revealed that the annual number of publications related to drug resistance and CRC climbed steadily. The number of publications on CRC drug resistance in 2021 was the highest in the last 2 decades (210), and it can be predicted that the number of publications will continue to rise steadily in the coming years according to the growth curve of publications over the years.

### Contribution of countries/regions to global publications

**Figure 3:**
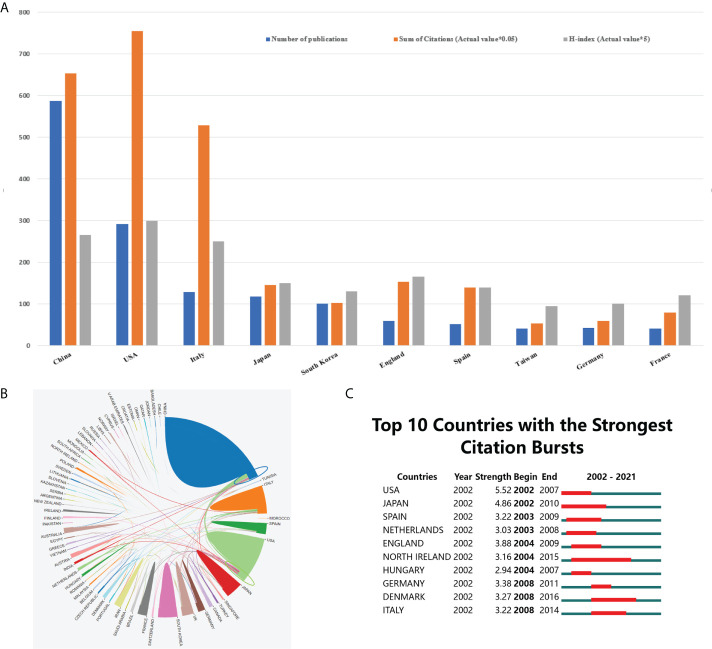
The contributions of different countries/regions to research concerning drug resistance and colorectal cancer. **(A)** The number of publications, citation frequency (×0.05), and H-index (×5) in the top 10 countries or regions; **(B)** The cooperation of countries/regions from 2002 to 2021; more lines emanating from a country indicate closer international cooperation; **(C)** Top 10 countries with the strongest citation bursts. The blue line represents the time axis, and the red portion on the blue time axis represents the interval at which the burst was found, including the start year, end year, and burst duration.

The annual trends of the top 5 countries with the most relevant publications in the field of CRC drug resistance from 2002 to 2021 were shown in **Figure 2B**. China ranked first with regard to the number of publications (587, 40.5%), followed by the USA (291, 20.0%), Italy (129, 8.9%), Japan (117, 8.1%) and South Korea (100, 6.9%). Except for China, the number of published articles in other countries has slowed down significantly in recent years, while China still shows a strong growth trend. The rest of top 10 countries/regions in terms of the total publications were illustrated in **Figure 3A**. Noteworthily, the top 5 countries with the highest centrality were Germany, Hungary, Finland, Denmark and Australia. Both the country and the ranking were completely different from the aforementioned top 5 countries with the most relevant publications. From the perspective of literature citation frequency, the top 5 countries were the USA (15100 times), China (13056 times), Italy (10575 times), England (3059 times) and Japan (2911 times). Despite China having the most publications, the H-index of China was 53, which was in second place after the USA (60).

In addition, we analyzed the collaborative level among countries/regions in this field and mapped out the cooperation links among countries/regions using the online platform of bibliometrics. As shown in **Figure 3B**, China and the USA had closer international cooperation than other countries/regions. Moreover, we analyzed the top 10 countries with the strongest citation bursts, as shown in **Figure 3C**, the USA, Japan and England were the countries with the highest citation strength, which reflected that these countries had a quite degree of influence in this research field.

### Contribution of institutions to research on drug resistance in colorectal cancer

**Figure 4:**
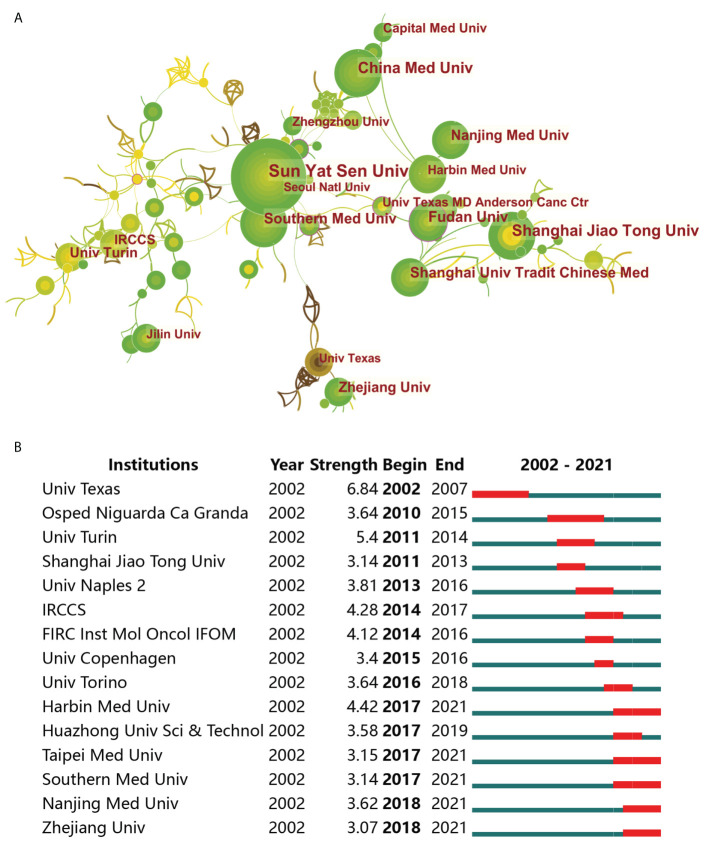
The contributions of different institutions associated with drug resistance and colorectal cancer. **(A)** Global institutions collaboration analysis. The nodes represent institutions, and the lines mean connection between them. The publication number is proportional to the size of nodes, and the thickness of the connecting line is proportional to the degree of cooperation. **(B)** Top 15 institutions with the strongest citation bursts. The blue line represents the time axis, and the red portion on the blue time axis represents the interval at which the burst was found, including the start year, end year, and burst duration.

Globally, Shanghai Jiao Tong University (46 papers, 3.17%) was the institution with the most literature published in this field over the last 20 years, followed by University of Texas System (42, 2.90%) from the USA and University of Turin (38, 2.62%) from Italy. As shown in **Figure 4A**, the Seoul National University ranked first in the world with a centrality of 0.21, despite having only 12 publications. Moreover, we analyzed the top 15 institutions with the strongest citation bursts, University of Texas System ranked first with a citation strength of 6.84. However, Harbin Medical University and Nanjing Medical University performed well in the citations of papers recently (**Figure 4B**). Of the top 10 institutions with respect to the number of articles published, five were from China, while the USA and Italy occupied two seats each, and remaining one was the Institut National de la Santé et de la Recherche Médicale from France (**Table 1**).

**Table 1:** Top 10 institutions published studies related to drug resistance in colorectal cancer.

Rank	Institutions	Country	Number of studies	Percentage (N/1451)
1	Sun Yat-sen University	China	46	3.17
2	University of Texas System	USA	42	2.90
3	University of Turin	Italy	38	2.62
4	Shanghai Jiao Tong University	China	35	2.41
5	Fondazione del Piemonte per l’ Oncologia-Istituto di Ricovero e Cura a Carattere Scientifico (IRCCS)	Italy	30	2.07
6	UT MD Anderson Cancer Center	USA	29	2.00
7	Southern Medical University China	China	26	1.79
8	Fudan University	China	25	1.72
9	Institut National de la Santéet de la Recherche Médicale	France	25	1.72
10	Shanghai University of Traditional Chinese Medicine	China	24	1.65

### Analysis of journals and co-cited journals

**Figure 5:**
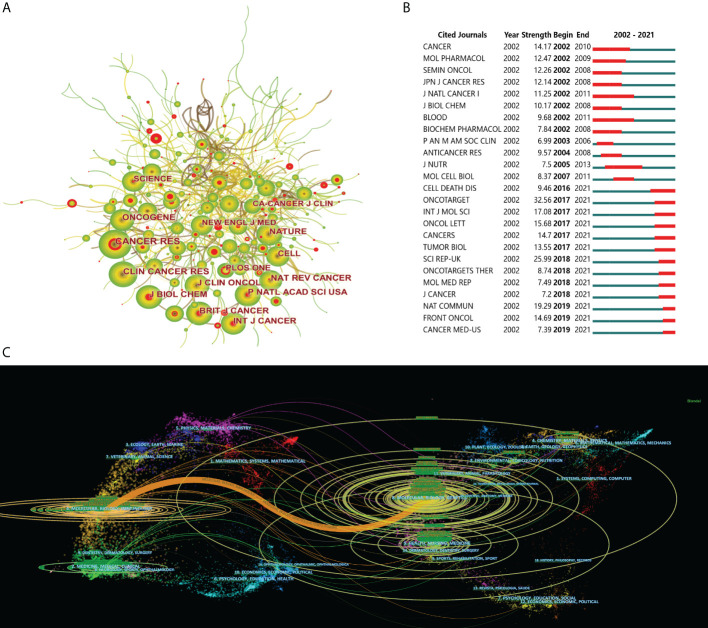
The contributions of journals in drug resistance and colorectal cancer. **(A)** Journals co-citation analysis. The nodes represent journals, and the lines mean citations between them. The publication number is proportional to the size of nodes, and the thickness of the connecting line is proportional to the degree of citations. The red core of the nodes represents stronger citation bursts. From 2002 to 2021, the color changed from yellow to green. **(B)** Top 25 cited journals with the strongest citation bursts. The blue line represents the time axis, and the red portion on the blue time axis The contributions of journals in drug resistance and colorectal cancer. **(C)** The dual-map overlay of journals related to colorectal cancer drug resistance. On the left were the citing journals, on the right were the cited journals, and the colored path represented the citation relationship.

A total of 307 articles, 21.2% of the whole literature, were published in the top 10 journals during the last 20 years. *Oncotarget* (49, 3.4%), *Clinical Cancer Research* (37, 2.6%), *Cancers* (34, 2.3%), *Cancer Letters* (31, 2.1%) and *Oncology Reports* (29, 2.0%) ranked in the top 5, accounting for 12.4% of all publications related to drug resistance and CRC (**Table 2**). Among the top ten most prolific journals, *Cancer Research* had the highest IF of 12.70. The analysis of co-cited journals related to drug resistance in CRC was illuminated in **Figure 5A**, unlike the top 10 most prolific journals, highly cited journals include the *New England Journal of Medicine*, *Nature*, and *Cell.* Moreover, we analyzed the top 25 journals with the strongest citation bursts, *Oncotarget* and *Scientific Reports* took the top two spots with a citation strength of 32.56 and 25.99. While in recent three years, publications in *Nature Communications, Frontiers in Oncology* and *Cancer Medicine* performed well in the number of citations (**Figure 5B**). Moreover, the dual-map overlay can reveal the citing situation of academic journals from various fields (11). As displayed in **Figure 5C**, in the field of drug resistance and CRC, researches published to journals of molecular/biology/genetics were primarily cited by researches published in molecular/biology/immunology.

**Table 2:** Top 10 productive journals related to drug resistance in colorectal cancer.

Rank	Journal	Studies counts	Percentage (N/1451)	IF
1	Oncotarget	49	3.38	Not Found
2	Clinical Cancer Research	37	2.55	12.53
3	Cancers	34	2.34	6.64
4	Cancer Letters	31	2.14	8.68
5	Oncology Reports	29	2.00	3.91
6	Oncology Letters	28	1.93	2.97
7	Cancer Research	25	1.72	12.70
8	International Journal of Molecular Sciences	25	1.72	5.92
9	International Journal of Oncology	25	1.72	5.65
10	Anticancer research	24	1.65	2.48

### Contribution of authors to research on drug resistance in colorectal cancer

**Figure 6:**
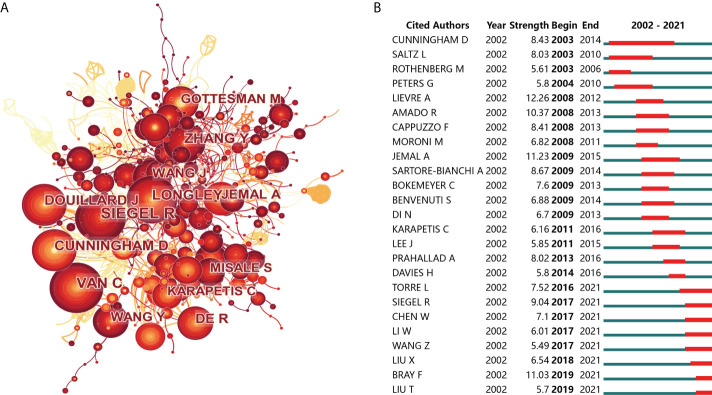
The contributions of authors in drug resistance and colorectal cancer. **(A)** Authors co-citation analysis. The nodes represent authors, and the lines mean citations between them. The publication number is proportional to the size of nodes, and the thickness of the connecting line is proportional to the degree of citations. **(B)** Top 25 authors with the strongest citation bursts. The blue line represents the time axis, and the red portion on the blue time axis represents the interval at which the burst was found, including the start year, end year, and burst duration.

No matter in the number of publications or citations, Bardelli A from University of Torino occupied the first place among all authors in the field of drug resistance in CRC, reflecting his remarkable contribution to the advance of this field. The second and third most prolific authors are Wang Y from China Pharmaceutical University and Zhang Y from University of Pennsylvania, with 27 and 21 publications, respectively. Of note, although Professor Di Nicolantonio F from the University of Turin only ranked fourth in the number of publications, he ranked second with 5,039 citations and an H-index of 19, second only to Professor Bardelli A. Half of the top 10 prolific authors were from Italy, and the other five were from the USA and China, which was consistent with the results of the top three high producing countries (**Table 3**). As indicated in **Figure 6A**, the co-cited author network presented that Siegel R (132 citations), Van C (106 citations) and Longley D (85 citations) were top three co-cited authors. Moreover, we analyzed the top 25 co-cited authors with the strongest citation bursts, Lievre A and Jemal A take the top two spots with a citation strength of 12.26 and 11.23. While in recent three years, Professor Bray F from France performed well in the number of citations and had been a rising star in the field (**Figure 6B**).

**Table 3:** Top 10 authors with most publications in research scope of drug resistance in colorectal cancer.

Rank	Author	Country	Affiliation	No. of Publications	No. of Citations	H-Index
1	Bardelli A	Italy	University of Torino	28	5971	24
2	Wang Y	China	China Pharmaceutical University	27	477	11
3	Zhang Y	USA	University of Pennsylvania	21	458	12
4	Di Nicolantonio F	Italy	University of Torino	20	5039	19
5	Wang X	China	Zhejiang University	18	445	12
6	Ciardiello F	Italy	University of Campania	17	662	13
7	Siena S	Italy	University of Milano	17	5028	15
8	Wang L	China	Southern Medical University	16	677	11
9	Zhang L	USA	University of Pittsburgh	16	672	13
10	Troiani T	Italy	University of Campania	15	541	10

### Analysis of cited reference related on drug resistance in colorectal cancer

**Figure 7:**
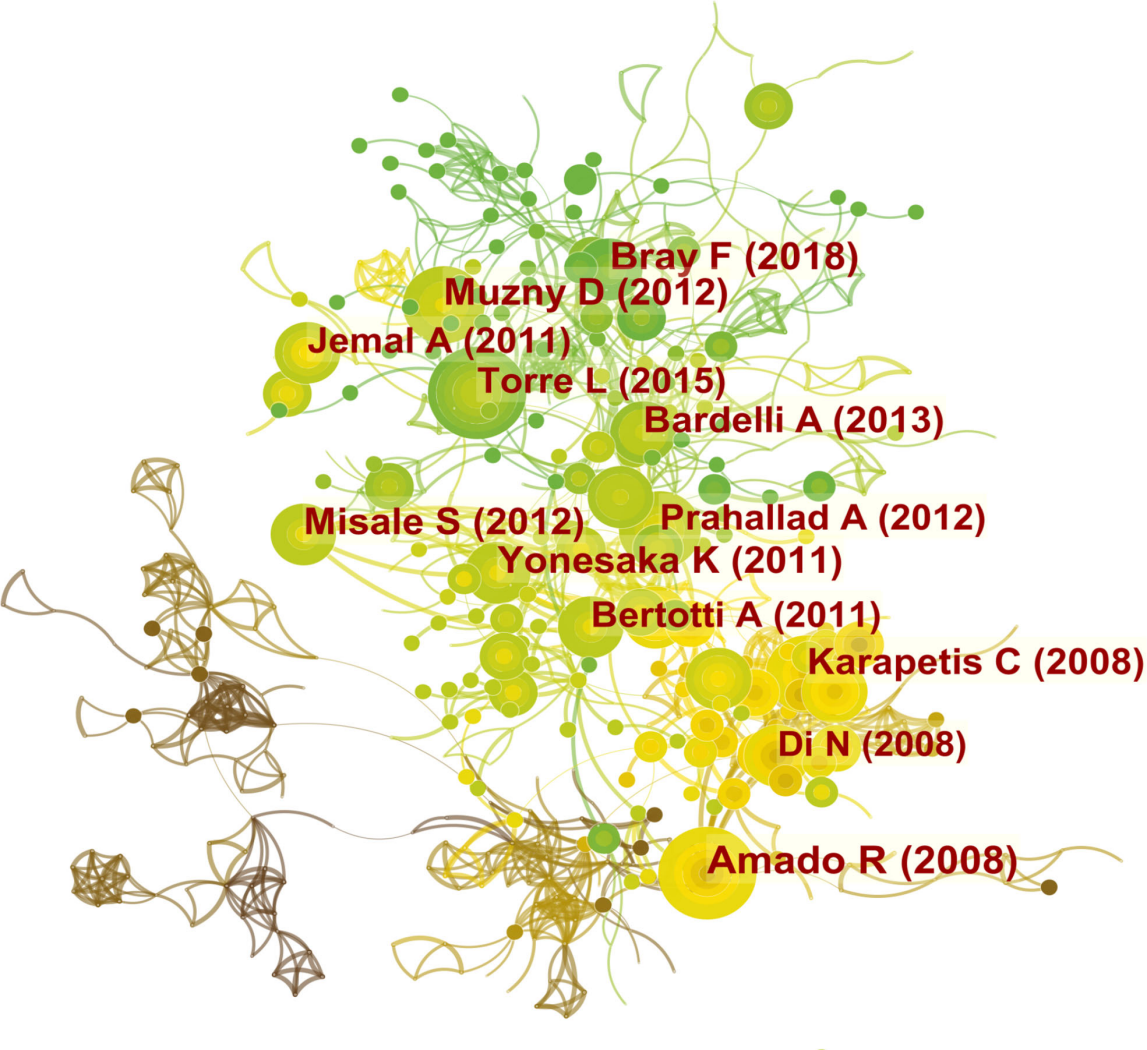
Network visualization diagram of cited references. Cited references are indicated by the node. The nodes represent cited references, and the lines mean citations between them. The citation number is proportional to the size of nodes, and the thickness of the connecting line is proportional to the degree of citations. From 2002 to 2021, the color changed from yellow to green.

In addition, we listed the top 10 articles in terms of frequency of citations, among which the most cited article entitled “Emergence of KRAS mutations and acquired resistance to anti-EGFR therapy in colorectal cancer” was conducted by Bardelli A et al. and published in *Nature* in 2012 (14). The total citation and average annual citation frequencies of this research were up to 1238 and 112.55, respectively. Regarding the top 10 papers, *Nature* and *Cancer Discovery* each published two articles, whereas the remaining 6 highly cited articles were published in distinct journals (**Table 4**). Noteworthily, 6 of the 10 high-cited papers were written by Bardelli A. **Figure 7** depicted the situation of co-cited literature in this field, and the co-cited literature network figure consisted of 831 nodes and 2971 links. By carefully reading the highly co-cited documents in the diagram, we could find that the result was basically consistent with the content of **Table 4**, except that the former showed the first author while the latter presented the corresponding authors.

**Table 4:** Top 10 high-cited papers related to drug resistance in colorectal cancer.

Title	Corresponding authors	Journal	Publication Year	Total Citations	Average per Year
Emergence of KRAS mutations and acquired resistance to anti-EGFR therapy in colorectal cancer	David Solit and Alberto Bardelli	NATURE	2012	1238	112.55
The molecular evolution of acquired resistance to targeted EGFR blockade in colorectal cancers	Luis A. Diaz Jr and Kelly S. Oliner	NATURE	2012	1184	107.64
Colon cancer stem cells dictate tumor growth and resist cell death by production of interleukin-4	Jan Paul Medema and Giorgio Stassi	CELL STEM CELL	2007	801	50.06
A Molecularly Annotated Platform of Patient-Derived Xenografts (Xenopatients) Identifies HER2 as an Effective Therapeutic Target in Cetuximab-Resistant Colorectal Cancer	Alberto Bardelli and Livio Trusolino	CANCER DISCOVERY	2011	617	51.42
PIK3CA Mutations in Colorectal Cancer Are Associated with Clinical Resistance to EGFR-Targeted Monoclonal Antibodies	Salvatore Siena and Alberto Bardelli	CANCER RESEARCH	2009	601	42.93
Assessment of somatic k-RAS mutations as a mechanism associated with resistance to EGFR-targeted agents: a systematic review and meta-analysis of studies in advanced non-small-cell lung cancer and metastatic colorectal cancer	Samuel Murray	LANCET ONCOLOGY	2008	595	39.67
Clonal evolution and resistance to EGFR blockade in the blood of colorectal cancer patients	Andrea Sartore-Bianchi and Alberto Bardelli	NATURE MEDICINE	2015	518	64.75
Molecular Mechanisms of Resistance to Cetuximab and Panitumumab in Colorectal Cancer	Alberto Bardelli	JOURNAL OF CLINICAL ONCOLOGY	2010	517	39.77
Amplification of the MET Receptor Drives Resistance to Anti-EGFR Therapies in Colorectal Cancer	Alberto Bardelli and Salvatore Siena	CANCER DISCOVERY	2013	455	45.5
KRAS codon 61, 146 and BRAF mutations predict resistance to cetuximab plus irinotecan in KRAS codon 12 and 13 wild-type metastatic colorectal cancer	F Loupakis	BRITISH JOURNAL OF CANCER	2009	435	31.07

### Analysis of keywords and research hotspots on drug resistance in colorectal cancer

**Figure 8:**
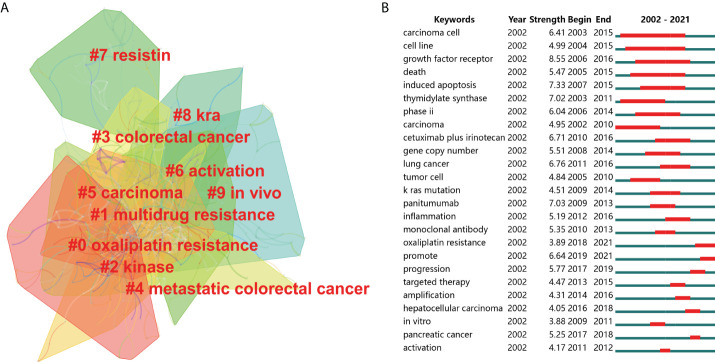
**(A)** The cluster of keywords related to colorectal cancer drug resistance. The different colors represented different clusters. **(B)** Top 25 keywords with the strongest citation bursts. The blue line represents the time axis, and the red portion on the blue time axis represents the interval at which the burst was found, including the start year, end year, and burst duration.

The network map of keywords was first built to visualize their clusters, as shown in **Figure 8A**, we divided the keywords in this filed into 10 clusters, namely “oxaliplatin resistance” (Cluster 0), “multidrug resistance” (Cluster 1), “kinase” (Cluster 2), “colorectal cancer” (Cluster 3), “metastatic colorectal cancer” (Cluster 4), “carcinoma” (Cluster 5), “activation” (Cluster 6), “resistin” (Cluster 7), “kra” (Cluster 8) and “*in vivo*” (Cluster 9). Moreover, we applied CiteSpace to conduct keywords burst analysis to determine the leading edges of research in this field during the past 2 decades. “Growth factor receptor” (burst strength of 8.55), “induced apoptosis” (burst strength of 7.33) and “panitumumab” (burst strength of 7.03) were the top 3 keywords with the highest burst strength in the past 15 years. In contrast, “promote” (burst strength of 6.64), “progression” (burst strength of 5.77) and “oxaliplatin resistance” (burst strength of 3.89) were the keywords with more occurrence in recent 5 years (**Figure 8B**).

**Figure 9:**
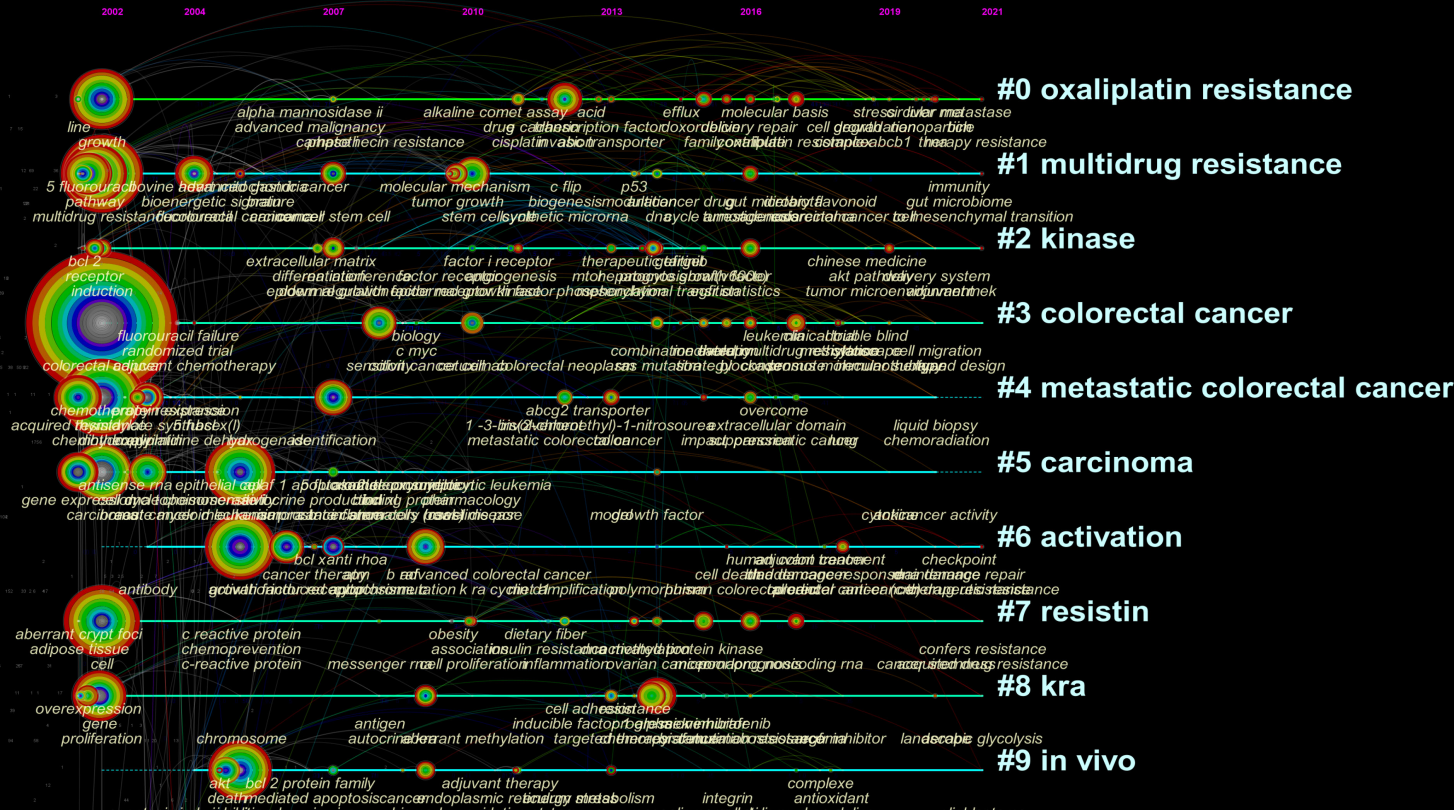
**A** timeline view for keywords associated with colorectal cancer drug resistance. The node’s position on the horizontal axis represents the time when the keyword first appeared, and the node’s size is positively correlated with the number of occurrences of the keywords. The lines between the nodes represent co-occurrence relationships. The redder the color means closer to 2021, and the grayer the color means closer to 2002.

**Figure 10:**
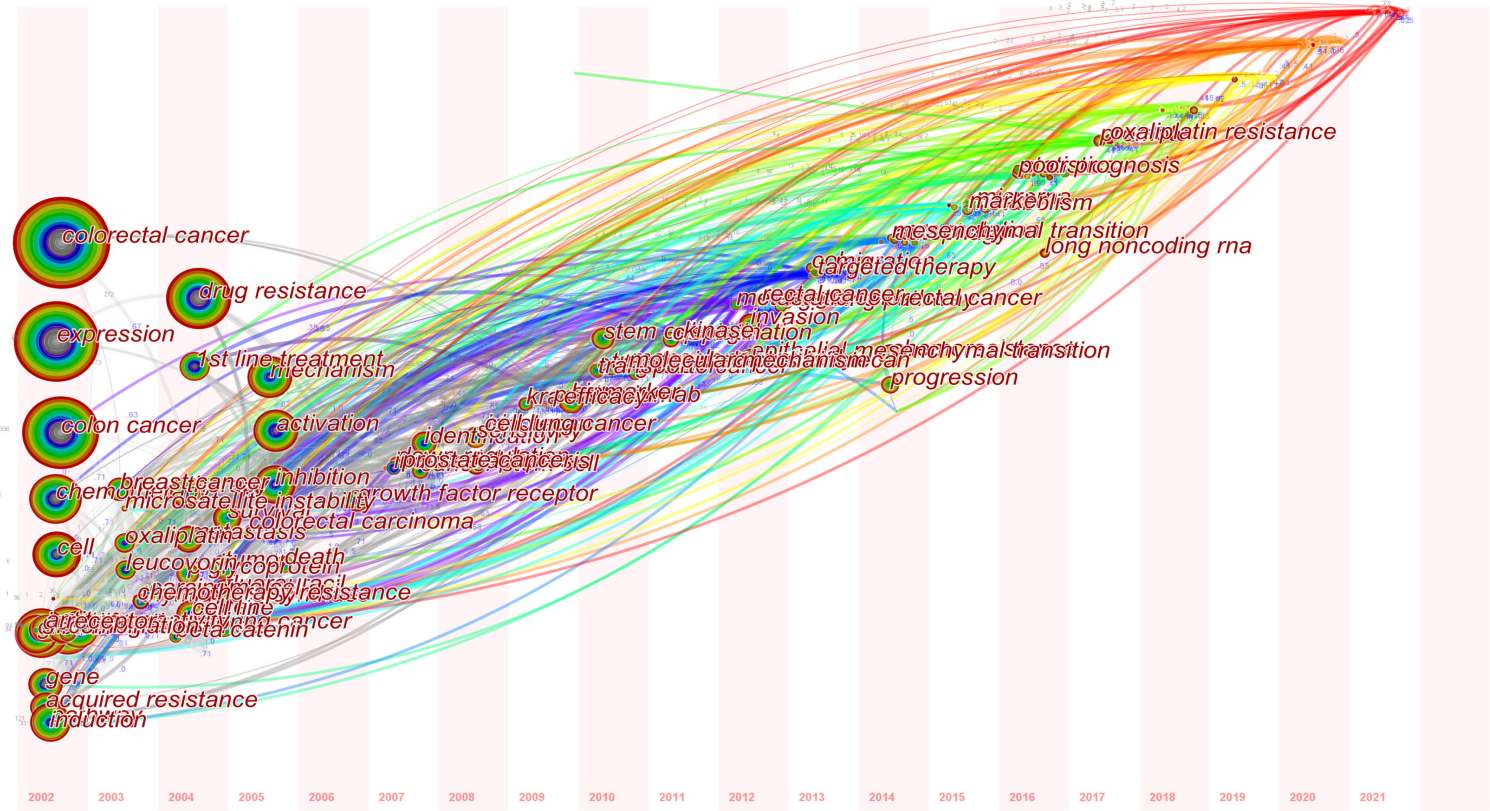
Time zone view of keywords (co-occurrence rate ≥40) for colorectal cancer drug resistance. The position of the circles on the horizontal axis represents the year in which the keyword first appeared. The size of the circle reflects the frequency of co-occurrence, and the lines between the circles represent the co-occurrence of two keywords.

Furthermore, we conducted a temporal analysis and outlined the chronology of keywords associated with drug resistance in CRC (**Figure 9**). The node’s location on the horizontal axis represented the time when the keyword first emerged, and the node’s dimensions was positively linked to the occurrence of keywords. The lines between the nodes represented keyword relationships. We found that “colorectal cancer” (Cluster 3), “multidrug resistance” (Cluster 1) and “metastatic colorectal cancer” (Cluster 4) were a relatively early hotspot, “carcinoma” (Cluster 5), “activation” (Cluster 6) and “*in vivo*” (Cluster 9) were a mid-term research hotspot. Keyword nodes contained in “Resistin” (Cluster 7), “KRA” (Cluster 8) and “oxaliplatin resistance” (Cluster 0) appeared late on the horizontal axis of time, demonstrating that these clusters were the novel research hotspots lately in this field. Similarly, the time zone diagram of high-frequency keywords (T > 40) realized by CiteSpace showed that “oxaliplatin resistance”, “long noncoding RNA” and “epithelial mesenchymal transition” were the latest research hotspots (**Figure 10**).

Finally, 137 keywords with at least 20 occurrence times were extracted from the 1451 eligible articles, and co-occurrence analysis was performed *via* VOSviewer. Detailed information on all included keywords is listed in **Supplemental Table 1**. During the early stage of the exploration of drug resistance and CRC, “cell-lines” (keyword AAY 2012.1), “phase-II” (keyword AAY 2012.1) and “induced apoptosis” (keyword AAY 2013.3) were the primary research focus. However, analysis of novel keywords revealed that “oxaliplatin resistance” (AAY 2019.3), “microRNAs” (AAY 2018.7) and “EMT” (AAY 2018.5) might be the research highlights in recent years, occurring 27, 30 and 27 times, respectively.

## Discussion

A total of 1451 publications with respect to drug resistance in CRC from 2002 to 2021 were obtained by searching the WOSCC. In terms of annual publications, we found that the publications in this area increased steadily each year, especially after 2017. China’s annual publications surged after 2011 and surpassed the USA for the first time in 2014, and has maintained the top spot ever since. The annual publications of Italy, Japan and South Korea were far less than China and the USA, and the growth rates of annual publications in the USA, Italy, Japan and South Korea have slowed down significantly in nearly five years, and even showed negative growth phenomenon. In contrast, the Chinese scholars stayed for continuing interests and attentions in this field, and the annual publications remained relatively substantial increase. It is conceivable that China’s record-breaking annual growth rate in this field is closely related to the financial support provided by Chinese government.

### Contribution analysis of countries/regions, institutions, journals and authors

Of note, China was not as far ahead in the academic impact of research in this field as it was in the volume of publications. With an H-index of 53, China was in second place behind the USA. Among the top 10 countries with the most publications over the past 20 years, the USA was the first to publish research in the field, which in part explained its extraordinary academic influence. Basic medical research in the USA seemed to have a superior environment and conditions, characterized by cutting-edge equipment, professional researchers, sufficient funds and extensive academic interaction. These strengths well explained why the USA become a leading force in this domain. Italy’s academic influence in this field was unquestionable, as evidenced by the fact that half of the top 10 prolific authors were from Italy and 8 of top 10 high-cited papers were produced by Italian researchers. Not only in China, a slight discrepancy between the quality and quantity of research also existed in South Korea. South Korea ranked fifth in terms of the total number of relevant studies, while its citation frequency and H-index ranked only seventh, behind the England and Spain. But it has to be said that South Korea’s performance in this field has been quite outstanding in being able to stand out among numerous developed countries. Although the high incidence of gastrointestinal tumors in South Korea provides an inherent advantage for domestic researchers to conduct research, the unremitting exploration of Korean researchers is also one of the indispensable factors for South Korea to be at the forefront of the world in this field. As **Figure 3B** shows, China and the USA had the most intense academic exchanges and cooperation with other countries. Within the context of global collaborative commons, an increasing number of important studies accomplished by multinational researchers working together will emerge in the future. Therefore, it is highly likely that future pioneering breakthroughs in this field will come in the form of cooperation between China or the United States and other countries.

Among the top 10 institutions in terms of the number of articles published, five were from China, while the USA and Italy occupied two seats each, which was in line with the leading position of these three countries in this field. Nine of the top 10 most frequently cited articles were published by the top 10 institutions, with six articles contributed by University of Turin in Italy. With a multitude of high-quality articles and institutions, Italy needs to improve international cooperation to jointly promote research associated with drug resistance and CRC worldwide. As a country with half of the top 10 productive institutions, China did not have a single top 10 cited article. The reasons behind this were thought-provoking, and it was urgent to improve the quality of Chinese scholars’ publications. Similarly, if the USA intends to continue its excellent academic standing in this area, more elite institutions will be required to participate in relevant research in the future.

Regarding the journals, *Oncotarget* (Not Found) published 49 relevant studies, far more than any other journals. Unfortunately, it was excluded from SCI, and it remains uncertain whether it will be re-included in the future. *Clinical Cancer Research* (IF= 12.53), *Cancers* (IF= 6.64) and *Cancer Letters* (IF= 8.58) were the other major journals publishing relevant articles, and we recommend researchers in the field to focus on these above-mentioned journals to keep track of hot topics and advances. Notably, publications in *Nature Communications* (IF= 14.92)*, Frontiers in Oncology* (IF= 6.24) and *Cancer Medicine* (IF= 4.45) performed well in the number of citations in recent three years, indicating that these journals were paying more and more attention and interest to the field of CRC drug resistance. Majority of the top 10 prolific journals were associated with oncology, and the dual-map overlay presented that the researches from molecular/biology/genetics co-cited journals were primarily cited by journals published in molecular/biology/immunology, which implied that the main direction of research related to CRC drug resistance is basic research represented by molecular mechanism and biological immunity.

Bardelli A from University of Torino was the one with most publications in research scope of drug resistance and CRC, manifesting his prominent contribution to the advance of this filed. Even more to the point, six of the top 10 most-cited papers were composed by professor Bardelli A. The leading two of his highly cited articles, “Emergence of KRAS mutations and acquired resistance to anti-EGFR therapy in colorectal cancer” and “A Molecularly Annotated Platform of Patient-Derived Xenografts (Xenopatients) Identifies HER2 as an Effective Therapeutic Target in Cetuximab-Resistant Colorectal Cancer”, were published in *Nature* (IF=49.96) and *Cancer Discovery* (IF=39.40), respectively. As a leader in CRC drug resistance research, professor Bardelli A focused on the molecular mechanisms and pharmacological efficacy of targeted drug resistance in CRC, especially anti-epidermal growth factor receptor (EGFR) drugs such as cetuximab and panitumumab. In addition to Bardelli A, there were four other authors from Italy among the top 10 prolific authors: Bardelli A’s colleagues Di Nicolantonio F from the University of Turin, Ciardiello F and Troiani T from the University of Campania, and Siena S from the University of Milan, which once again confirmed that great academic influence of Italy in this field.

### High-cited publications related to drug resistance and colorectal cancer

The articles with the leading citation frequency have a huge academic influence on research in certain fields. Details of the top 10 cited publications are shown in **Table 4**. EGFR therapy in colorectal cancer” has been cited 1238 times since its publication and is the most frequently quoted research about drug resistance and CRC. This research was published in *Nature* (IF=49.96) in 2012, and its corresponding authors were Solit D and Bardelli A from Italy (14). In this study, Sandra Misale et al. found that KRAS mutation was a frequent driver of acquired resistance to cetuximab in CRC, and the emergence of KRAS-mutated clones could be noninvasively detected months before radiographic progression. Since combination therapy of anti-EGFR and anti-mitogen-activated protein kinase (MEK) remained effective in those cetuximab-resistant cells, Sandra Misale et al. recommended early initiation of MEK inhibitors as a reasonable strategy to delay or reverse drug resistance. This study was the first to explore the molecular mechanism of acquired resistance to cetuximab in CRC and creatively proposed dynamic monitoring of KRAS mutations in CRC patients. Anti-MEK combination therapy guided by dynamic monitoring of ERAS mutations was expected to delay disease progression in cetuximab-resistant CRC patients.

The second and third most highly quoted studies explored molecular mechanism of the anti-EGFR and chemotherapeutic drug resistance in CRC, which were published in *Nature* (IF=49.96) and *Cell Stem Cell* (IF=24.63), respectively. Unlike the above study conducted by Sandra Misale, although Luis A. Diaz Jr’s team also focused on the acquired resistance to anti-EGFR therapy in CRC, the latter was achieved by testing 28 patients who received panitumumab monotherapy and confirmed that KRAS mutation was an important mechanism of acquired resistance to anti-EGFR therapy with the clinical view. What’s more, this team proposed that acquired resistance to anti-EGFR therapy was a fait accompli and that the timing of the emergence of resistance depended on the time interval required for tumor cell subclones to repopulate the lesion (15). Another study entitled “Colon cancer stem cells dictate tumor growth and resist cell death by production of interleukin-4” was completed by Matilde Todaro et al. in 2007, this study used colon cancer stem cells to demonstrate that CD133^+^-expressing colon cancer stem cells could produce and utilize IL-4 to protect themselves from apoptosis, thereby resisting chemotherapy. Consistently, administering IL-4Ra antagonists or anti-IL-4 neutralizing antibodies strongly augmented the antitumor efficacy of standard chemotherapeutics (16). This study provided a new intervention target for reversing CRC chemotherapy resistance, which had great clinical significance. Taken together, the majority of the top 10 most cited articles focused on exploring the mechanisms behind the link between CRC and drug resistance, especially anti-EGFR monoclonal antibodies such as cetuximab and panitumumab.

### Keywords analysis of drug resistance in colorectal cancer

Keywords analysis includes burst analysis, temporal analysis and AAY analysis, which can intuitively reflect the time of the emergence of the keyword in the early or late and the novelty degree. By comprehensively considering these keyword analysis results, we found that “growth factor receptor”, “panitumumab” and “induced apoptosis” were the research hotspots in the early stage of this field. Radical surgical resection, chemotherapy and radiotherapy are the most classical methods of tumor treatment, while conventional chemotherapy or radiotherapy may cause a series of side effects while killing tumor cells (17). Japanese scholar Kawashima S et al. found that inhibition of γ H2A histone family member X (γ-H2AX) enhanced sensitivity during radiotherapy, and γ-H2AX showed potential as a new predictor of the efficacy and resistance of preoperative radiotherapy in CRC patients (18). With the development of molecular biology technology and the in-depth understanding of tumor pathogenesis from the molecular level of cell receptor and proliferation regulation, molecular targeted therapy has entered the eyes of researchers and gradually became a new favorite due to its specificity, pertinence and effectiveness (19). Growth factor (GF) is a kind of polypeptide material that regulates cell growth and other cell functions by binding with specific and high affinity cell membrane receptor. At present, known GFRs include EGFR, insulin-like growth factor receptor (IGFR), vascular endothelial growth factor receptor (VEGFR) and so on (20). Previous studies have shown that oncogenes or oncoproteins can lead to uncontrolled cell proliferation and carcinogenesis by interfering with the intercellular signaling pathways of GFs and their receptors (21). In the field of metastatic CRC, EGFR-targeted small molecule inhibitors or monoclonal antibodies have gradually become the mainstream of targeted therapy (22). EGFR is a member of the tyrosine kinase type I receptor subfamily, other members of which include HER2/NEU, HER3 and HER4 (23). Activated EGFR is mainly associated with the following signaling channels: mitogen-activated protein kinase/extracellular signal-regulated kinase (MAPK/ERK) pathway, phosphatidylinositol 3-kinase/protein kinase B (PI3K/Akt) pathway and epithelial growth factor receptor-signal transduction and transcription activator 3(EGFR-STAT3) pathway (24, 25). Therefore, EGFR can regulate the activity of a variety of genes and participate in the occurrence, development and apoptosis and other physiological processes of tumors. Although anti-RGFR monoclonal antibody therapy represented by cetuximab and panitumumab has achieved expected clinical efficacy, most CRC patients develop varying degrees of drug resistance after 5-8 months of treatment, which significantly reduces the clinical benefit of anti-RGFR therapy (26). Since then, experts in this field have shifted their research focus to the molecular mechanism and prevention strategies of anti-EGFR drug resistance. Recently, the molecular mechanisms of anti-RGFR drug resistance that have been elucidated include KRAS, BRAF, PIK3CA, and PTEN gene mutations, and MET receptor amplification, etc (4, 7). Correspondingly, abnormal changes in the above genes can be used as biomarkers for predicting anti-EGFR drug resistance, and early detection of these abnormal changes can help to screen out drug-resistant patients and start combination therapy as soon as possible.

Another research hotspot in the early stage of this field was “induced apoptosis”. Under the drive of proto-oncogene, abnormal proliferation and reduced apoptosis of normal cells are important molecular mechanisms to induce carcinogenesis. Correspondingly, the ultimate goal of most anticancer drugs in clinical practice is to induce selective cell death of tumor cells, and apoptosis is an important part of it. Unfortunately, while these drugs induce tumor cell apoptosis, they also force tumor cells to undergo adaptive changes such as mutations of antiapoptotic genes or the generation of drug resistance genes. Although numerous studies on chemotherapy resistance have been conducted in the past 20 years, there seems to be not much substantial progress (27, 28). The currently accepted way to circumvent or delay the occurrence of chemotherapy resistance is to combine chemotherapy with other drugs, such as immune checkpoint inhibitors or anti-EGFR targeted drugs.

In addition to revealing early research hotspots in CRC drug resistance, we also predicted potential future research directions in this field through keyword analysis. Analysis of novel keywords presented that “oxaliplatin resistance”, “microRNA” and “EMT” were recently appeared frontiers of research that should be closely tracked in the future. As a third-generation platinum medicine, oxaliplatin is widely exploited for the therapy of CRC (29). Bonetti A et al. found that oxaliplatin/5-fluorouracil (5FU)/leucovorin (LV) combination provides a significant improvement of disease control in advanced CRC versus 5FU/LV alone (30). Unfortunately, oxaliplatin treatment also has the same inevitable phenomenon of acquired resistance as other anti-tumor drugs. The molecular mechanisms of oxaliplatin resistance that have been elucidated included variations of oxaliplatin delivery system, detoxification of oxaliplatin, abnormality of DNA damage response and repair, attenuated cell death (apoptotic and nonapoptotic), and so on (31, 32). Moreover, drug resistance of 5-FU treatment is also a non-negligible problem. Recent studies have found that sphingomyelin and ceramide play an important role in this process. Sphingomyelin, ceramide and acid sphingomyelinase may be novel potential target molecules to overcome 5-FU resistance (33). How to translate these molecular mechanisms and biomarkers of drug resistance into clinical benefits for CRC patients will undoubtedly be the focus of future research in this field (34).

EMT is the course that cells lose their epithelial properties and acquire mesenchymal properties, which was first described in embryogenesis. It’s well known that EMT is a multifaceted and reversible biological process involving cellular, genetic, physiological, metabolic and any other changes. Moreover, EMT is closely related to the progression, metastasis drug resistance of CRC. Growing evidence from preclinical and early clinical studies suggests that EMT endows tumor cells with the ability to adapt to changing microenvironments during tumor progression, thereby further enhancing tumor cell resistance to chemotherapy, radiotherapy, targeted therapy and even immunotherapy (35). In addition, studies have confirmed that the up-regulation of Notch-1, HES1 and HEY1 genes, overexpression of *Snail* gene and the aberrant initiation of Wnt/β-catenin signaling pathway are strongly associated with the EMT of CRC cells and resistance to cisplatin drugs (36, 37). Therefore, how to deeply explore the molecular mechanism of CRC drug resistance from the perspective of EMT and combine it with clinical practice will be another research hotspot in the future.

MicroRNAs (miRNAs) refer to a class of non-coding single-stranded small RNAs (18-22 nucleotides) (38). A body of evidences proved that miRNAs play a critical part in EMT-related metastasis and drug resistance of tumor cells (39). For example, miR-139-5p and miR195-5p remarkably inhibited the metastatic capacity and chemoresistance of CRC by targeting BCL2 and glycerophosphodiester phosphodiesterase domain containing 5 (GDPD5), respectively (40, 41). Victoria J Findlay discovered that SNAI2 regulated CRC 5-fluorouracil sensibility through inhibiting miR145, and proposed that the SNAI2/miR145 pathway may be a response predictor and new clinical therapeutic target in CRC chemotherapy (42). To sum up, targeting miRNAs to antagonize certain malignant properties of tumors may have broader clinical implications. Additionally, the expression of miRNAs in CRC is comprehensively modulated by splicing factors, whose dysregulation is found to be a significant modulator for treatment resistance in CRC (43). Noteworthily, serine and arginine rich splicing factor (SRSF) families, including SRSF1, SRSF3 and SRSF6, are the most thoroughly studied splicing factors in recent years. These factors may become new therapeutic targets for inhibiting CRC progression or even reversing drug resistance (44–46). Further elucidation of the molecular pathways by which microRNAs affect drug resistance in CRC will be another potential focus of current and future research in this field.

### Strengths and limitations

We conducted a comprehensive and objective analysis of research developments in the field of drug resistance and CRC over the past 20 years. Moreover, the analysis and prediction of possible future research hotspots were carried out from the perspective of bibliometrics, which could provide some reference for relevant research. Nonetheless, some limitations are still inevitable. First, only English articles were enrolled, which means that some potentially valuable non-English literature was ignored and excluded from our research. Second, the types of research that met the inclusion criteria were limited to articles and reviews. Thus, letters, conference papers and books, which may have academic impact, were excluded. Third, literature published before 2002 could not be found in the current research.

## Conclusions

Taken together, this study summarizes the global research status of drug resistance and CRC during 2002-2021. China published the most articles, and the USA had the highest H-index, while Italy had more outstanding elite researchers in this important field. Updates on the latest research or advances can be found in *Oncotarget*, *Clinical Cancer Research* and *Cancers.* Previous studies have mainly concentrated on growth factor receptor and induced apoptosis. Oxaliplatin resistance, microRNA and EMT as recently appeared frontiers of research that should be closely tracked in the future.

## Data availability statement

The original contributions presented in the study are included in the article/**Supplementary Material**. Further inquiries can be directed to the corresponding authors.

## Author contributions

X-HD and P-YZ conceptualized, supervised, and edited the manuscript. P-YZ, Y-NJ and Z-FM extracted all data and performed the bibliometric analyses. YY, Y-XL, S-DH and S-YL undertook and refined the searches. P-YZ and Y-NJ codrafted the paper. All authors contributed to the article and approved the submitted version.

## Funding

This work was supported by grants from the National Natural Science Foundation of China (No. 81871317) and the Key Project of Military Medical Innovation Program (No. 18CXZ025).

## Conflict of interest

The authors declare that the research was conducted in the absence of any commercial or financial relationships that could be construed as a potential conflict of interest.

## Publisher’s note

All claims expressed in this article are solely those of the authors and do not necessarily represent those of their affiliated organizations, or those of the publisher, the editors and the reviewers. Any product that may be evaluated in this article, or claim that may be made by its manufacturer, is not guaranteed or endorsed by the publisher.

## Abbreviations

CRC, colorectal cancer; WOSCC, Web of Science Core Collection; EMT, epithelial-mesenchymal transition; AAY, average appearing year; ABC, ATP-binding cassette; MRPs, multidrug resistance proteins; SCI-E, Science Citation Index-Expanded; TI, title; IF, impact factor; JCR, Journal Citation Reports; EGFR, epidermal growth factor receptor; MEK, mitogen-activated protein kinase; γ-H2AX, γ H2A histone family member X; GF, growth factor; GFR, growth factor receptor; IGFR, insulin-like growth factor receptor; VEGFR,vascular endothelial growth factor receptor; MAPK/ERK, mitogen-activated protein kinase/extracellular signal-regulated kinase; PI3K/Akt, phosphatidylinositol 3-kinase/protein kinase B; STAT3, signal transduction and transcription activator 3; 5FU, 5-fluorouracil; LV, leucovorin; miRNAs, MicroRNAs; GDPD5, glycerophosphodiester phosphodiesterase domain containing 5; SRSF, serine and arginine rich splicing factor.
