# Supplementary material for: Publication trends and hotspots of drug resistance in colorectal cancer during 2002-2021: A bibliometric and visualized analysis
Source: Front Oncol. 2022 Aug 30;12:947658. doi: 10.3389/fonc.2022.947658 (PMC9469653; doi:10.3389/fonc.2022.947658)
Supplement: Supplementary file 1 [file Table_1.doc]

| Rank | Keywords | Cluster | Links | Occurrences | Average appearing years (AAY) | | Average citations |
| --- | --- | --- | --- | --- | --- | --- | --- |
| 1 | 1st-line treatment | 2 | 101 | 73 | 2014.9589 | 59.726 | |
| 2 | 5-fluorouracil | 1 | 119 | 157 | 2015.121 | 23.6752 | |
| 3 | 5-fu | 5 | 85 | 38 | 2016.3684 | 17.8947 | |
| 4 | 5-fu resistance | 1 | 65 | 23 | 2017.3478 | 33.4783 | |
| 5 | abcg2 | 4 | 52 | 23 | 2016.913 | 27.3913 | |
| 6 | acquired-resistance | 2 | 100 | 81 | 2016.7901 | 52.9012 | |
| 7 | activation | 1 | 129 | 152 | 2016.1133 | 28.6053 | |
| 8 | akt | 3 | 68 | 24 | 2017.3333 | 23.9167 | |
| 9 | amplification | 2 | 62 | 23 | 2015.2174 | 50.7391 | |
| 10 | angiogenesis | 1 | 82 | 32 | 2015.75 | 31.25 | |
| 11 | antitumor-activity | 2 | 83 | 36 | 2013.8889 | 37.8889 | |
| 12 | apoptosis | 1 | 131 | 252 | 2015.2698 | 25.4365 | |
| 13 | autophagy | 1 | 97 | 50 | 2017.76 | 27.36 | |
| 14 | bcl-2 | 1 | 64 | 20 | 2014.15 | 35.75 | |
| 15 | beta-catenin | 1 | 80 | 43 | 2015.4419 | 36.186 | |
| 16 | bevacizumab | 2 | 78 | 30 | 2016.7333 | 43.2 | |
| 17 | biomarkers | 2 | 77 | 27 | 2017.56 | 14.2593 | |
| 18 | blockade | 2 | 51 | 20 | 2018.9 | 32.2 | |
| 19 | braf | 2 | 64 | 35 | 2015.8286 | 48.1714 | |
| 20 | breast-cancer | 3 | 119 | 93 | 2015.7419 | 46.5161 | |
| 21 | cancer stem cells | 1 | 66 | 26 | 2017 | 29.1538 | |
| 22 | carcinoma | 3 | 120 | 103 | 2014.9223 | 27.7087 | |
| 23 | carcinoma cells | 1 | 52 | 20 | 2012.35 | 40.4 | |
| 24 | cell lung-cancer | 2 | 65 | 27 | 2015.1111 | 60.9259 | |
| 25 | cell-lines | 1 | 66 | 23 | 2012.1304 | 30.6957 | |
| 26 | cells | 3 | 126 | 176 | 2017.289 | 39.5852 | |
| 27 | cetuximab | 2 | 96 | 90 | 2016.6404 | 67.2889 | |
| 28 | cetuximab plus irinotecan | 2 | 45 | 27 | 2014.2593 | 64.6667 | |
| 29 | chemoresistance | 3 | 123 | 152 | 2017.298 | 24.1316 | |
| 30 | chemotherapy | 2 | 132 | 205 | 2015.4118 | 34.9854 | |
| 31 | chemotherapy resistance | 5 | 67 | 26 | 2016.6923 | 19.6154 | |
| 32 | cisplatin | 4 | 86 | 47 | 2014.5745 | 23.9787 | |
| 33 | colon | 2 | 81 | 40 | 2017.125 | 25.25 | |
| 34 | colon cancer | 1 | 128 | 177 | 2015.6497 | 22.9153 | |
| 35 | colon-cancer | 2 | 128 | 162 | 2015.7702 | 31.037 | |
| 36 | colorectal cancer | 2 | 134 | 481 | 2017.1234 | 20.5094 | |
| 37 | colorectal carcinoma | 1 | 76 | 27 | 2014.6667 | 29 | |
| 38 | colorectal-cancer | 1 | 125 | 149 | 2015.0201 | 29.8591 | |
| 39 | combination | 2 | 84 | 34 | 2015.0294 | 31.9412 | |
| 40 | crc | 3 | 67 | 22 | 2018.5 | 13.5909 | |
| 41 | cytotoxicity | 4 | 55 | 28 | 2014.0714 | 17.5714 | |
| 42 | death | 1 | 87 | 47 | 2014.1702 | 26.0426 | |
| 43 | differentiation | 3 | 69 | 22 | 2015 | 72 | |
| 44 | dna | 2 | 66 | 23 | 2015.4348 | 90.4783 | |
| 45 | down-regulation | 3 | 92 | 45 | 2016.3864 | 27.3111 | |
| 46 | doxorubicin | 4 | 63 | 34 | 2016.6765 | 23.6471 | |
| 47 | drug resistance | 4 | 130 | 163 | 2016.8834 | 22.7669 | |
| 48 | drug-resistance | 4 | 126 | 134 | 2016.6742 | 25.694 | |
| 49 | efficacy | 2 | 67 | 27 | 2016.2963 | 51.2222 | |
| 50 | egfr | 2 | 84 | 45 | 2015.8667 | 26.5778 | |
| 51 | emt | 3 | 69 | 27 | 2018.5385 | 11.7778 | |
| 52 | epithelial-mesenchymal transition | 3 | 99 | 62 | 2017.8871 | 24.9516 | |
| 53 | expression | 1 | 134 | 408 | 2016.1376 | 27.8578 | |
| 54 | family | 4 | 67 | 22 | 2015.9091 | 32.2273 | |
| 55 | fluorouracil | 2 | 109 | 75 | 2015.4133 | 33.04 | |
| 56 | gastric-cancer | 3 | 87 | 40 | 2015.85 | 37.075 | |
| 57 | gene | 1 | 107 | 69 | 2015.1449 | 36.5072 | |
| 58 | gene copy number | 2 | 36 | 20 | 2013.3 | 168.9 | |
| 59 | gene-expression | 1 | 104 | 66 | 2014.197 | 40.4394 | |
| 60 | genes | 4 | 61 | 20 | 2015.8 | 24.9 | |
| 61 | growth | 3 | 127 | 129 | 2016.3906 | 38.3721 | |
| 62 | growth-factor receptor | 2 | 83 | 53 | 2014.5849 | 62.3585 | |
| 63 | hepatocellular-carcinoma | 3 | 73 | 22 | 2016.4545 | 24.6818 | |
| 64 | hypoxia | 1 | 63 | 23 | 2015.3913 | 25.4348 | |
| 65 | identification | 2 | 98 | 64 | 2015.75 | 40.6094 | |
| 66 | in-vitro | 4 | 109 | 70 | 2015.5507 | 25.6143 | |
| 67 | in-vivo | 1 | 57 | 21 | 2011.5238 | 32.9048 | |
| 68 | induced apoptosis | 1 | 82 | 37 | 2013.3243 | 31.6757 | |
| 69 | induction | 1 | 78 | 38 | 2015.0263 | 39.0263 | |
| 70 | inflammation | 1 | 58 | 26 | 2015.3462 | 22.7308 | |
| 71 | inhibition | 1 | 124 | 115 | 2015.9826 | 30.3913 | |
| 72 | inhibitor | 4 | 72 | 33 | 2015.1212 | 19.7576 | |
| 73 | inhibitors | 4 | 61 | 22 | 2015.5714 | 20.5 | |
| 74 | invasion | 3 | 92 | 69 | 2018.2059 | 23.4928 | |
| 75 | irinotecan | 2 | 90 | 56 | 2014.0357 | 23.8571 | |
| 76 | kinase | 1 | 70 | 28 | 2015.2143 | 46.3571 | |
| 77 | kras | 2 | 74 | 57 | 2015.6491 | 79.9298 | |
| 78 | leucovorin | 2 | 83 | 44 | 2014.3409 | 45.0227 | |
| 79 | lines | 4 | 63 | 20 | 2012.3 | 35.9 | |
| 80 | lung-cancer | 2 | 93 | 52 | 2014.8462 | 73.3462 | |
| 81 | mechanism | 4 | 86 | 30 | 2016.2333 | 37.8 | |
| 82 | mechanisms | 4 | 119 | 127 | 2016.619 | 33.6457 | |
| 83 | mesenchymal transition | 3 | 72 | 26 | 2018.28 | 31.1538 | |
| 84 | metabolism | 1 | 81 | 30 | 2017.5 | 23.0667 | |
| 85 | metastasis | 3 | 115 | 135 | 2016.8271 | 27.6667 | |
| 86 | metastatic colorectal cancer | 2 | 55 | 20 | 2015.6 | 23.1 | |
| 87 | microrna | 4 | 72 | 26 | 2017.0385 | 40.4231 | |
| 88 | micrornas | 3 | 70 | 30 | 2018.6897 | 26.5667 | |
| 89 | microsatellite instability | 2 | 59 | 28 | 2014.2143 | 79 | |
| 90 | migration | 3 | 70 | 28 | 2017.7143 | 19.6786 | |
| 91 | molecular-mechanisms | 1 | 74 | 26 | 2017.2692 | 22.7692 | |
| 92 | multidrug resistance | 4 | 92 | 73 | 2014.7671 | 22.5342 | |
| 93 | multidrug-resistance | 4 | 113 | 101 | 2015.5149 | 32.5446 | |
| 94 | mutations | 2 | 88 | 54 | 2016.5185 | 56.7407 | |
| 95 | nf-kappa-b | 1 | 83 | 31 | 2014.5806 | 26.0968 | |
| 96 | ovarian-cancer | 3 | 66 | 21 | 2017.381 | 28.9524 | |
| 97 | overexpression | 3 | 98 | 43 | 2016.9302 | 25.3023 | |
| 98 | oxaliplatin | 2 | 124 | 124 | 2016.0161 | 23.9435 | |
| 99 | oxaliplatin resistance | 3 | 68 | 27 | 2019.2593 | 11.6667 | |
| 100 | p-glycoprotein | 4 | 90 | 86 | 2014.5059 | 25.7674 | |
| 101 | p53 | 1 | 77 | 25 | 2015.56 | 30.8 | |
| 102 | panitumumab | 2 | 67 | 38 | 2015.0263 | 103.6053 | |
| 103 | pathway | 1 | 122 | 106 | 2016.6762 | 32.8679 | |
| 104 | phase-ii | 2 | 60 | 24 | 2012.0833 | 41.625 | |
| 105 | phase-iii trial | 2 | 44 | 22 | 2015.1364 | 114.8182 | |
| 106 | phosphorylation | 1 | 68 | 27 | 2015.8148 | 31.963 | |
| 107 | poor-prognosis | 3 | 71 | 26 | 2018.3077 | 31.9231 | |
| 108 | prognosis | 3 | 84 | 34 | 2017.7941 | 16.4706 | |
| 109 | progression | 3 | 100 | 54 | 2017.2222 | 26.537 | |
| 110 | proliferation | 3 | 111 | 112 | 2017.3839 | 18.6696 | |
| 111 | promotes | 3 | 67 | 31 | 2018.6452 | 30.7097 | |
| 112 | prostate-cancer | 3 | 80 | 30 | 2015.1 | 36.0667 | |
| 113 | protein | 1 | 99 | 58 | 2016.1034 | 22.4138 | |
| 114 | proteins | 1 | 54 | 22 | 2016.4091 | 30.6818 | |
| 115 | radiotherapy | 1 | 56 | 23 | 2016.2174 | 16.3043 | |
| 116 | ras | 2 | 64 | 22 | 2015.7619 | 27.0909 | |
| 117 | ras mutations | 2 | 53 | 26 | 2017.6538 | 21.6538 | |
| 118 | receptor | 1 | 73 | 28 | 2015 | 54.5714 | |
| 119 | rectal cancer | 1 | 54 | 25 | 2015.28 | 20.32 | |
| 120 | resistance | 2 | 116 | 77 | 2016.7143 | 19.8052 | |
| 121 | risk | 1 | 42 | 21 | 2014.1905 | 37.3333 | |
| 122 | sensitivity | 4 | 101 | 54 | 2016.1111 | 21.2778 | |
| 123 | signaling pathway | 3 | 77 | 27 | 2016.8148 | 20.8889 | |
| 124 | statistics | 1 | 88 | 32 | 2018.4375 | 14.5625 | |
| 125 | stem-cells | 1 | 100 | 62 | 2017.0806 | 27.9516 | |
| 126 | suppression | 1 | 64 | 21 | 2016.4762 | 32.9048 | |
| 127 | survival | 2 | 117 | 87 | 2016.4138 | 26.908 | |
| 128 | target | 1 | 62 | 22 | 2017.9091 | 13.5455 | |
| 129 | therapy | 2 | 125 | 117 | 2016.6983 | 41.6325 | |
| 130 | thymidylate synthase | 4 | 82 | 40 | 2012.9 | 35.95 | |
| 131 | transporters | 4 | 60 | 22 | 2015.2273 | 30.3182 | |
| 132 | tumor | 1 | 85 | 35 | 2015.6 | 23.7429 | |
| 133 | tumor-cells | 4 | 55 | 21 | 2013.0952 | 38.8571 | |
| 134 | tumor-growth | 4 | 88 | 32 | 2016.2188 | 26.9688 | |
| 135 | tumorigenesis | 3 | 68 | 28 | 2017.4286 | 20.6429 | |
| 136 | tumors | 2 | 74 | 32 | 2013.5 | 71.9375 | |
| 137 | up-regulation | 3 | 79 | 32 | 2016.7188 | 26.625 | |

**Table S1** The analytic consequence of 137 keywords with at least 20 occurrence times
